# Characterization of retinal pigment epithelium layer in healthy and diseased retinas with high‐resolution adaptive optics transscleral flood illumination imaging

**DOI:** 10.1111/aos.70016

**Published:** 2025-10-07

**Authors:** Leila Sara Eppenberger, Safa Mohanna, Sohrab Ferdowsi, Sonja Simon‐Zoula, Oliver Pfäffli, Christoph Amstutz, Lucas M. Bachmann, Michael A. Thiel, Martin K. Schmid

**Affiliations:** ^1^ Eye Clinic Cantonal Hospital of Lucerne Lucerne Switzerland; ^2^ Department of Ophthalmology, Inselspital Bern University Hospital Berne Switzerland; ^3^ Singapore Eye Research Institute Singapore Singapore; ^4^ EarlySight SA Geneva Switzerland; ^5^ Epidemiology, Biostatistics & Prevention Institute (EBPI) University of Zurich Zurich Switzerland; ^6^ Medignition Inc Zurich Switzerland; ^7^ Faculty of Medicine University of Zurich Zurich Switzerland; ^8^ Faculty of Health Sciences and Medicine University of Lucerne Lucerne Switzerland

**Keywords:** adaptive optics transscleral flood illumination imaging, age‐related macular degeneration, in vivo quantitative imaging, retinal pigment epithelium imaging

## Abstract

**Purpose:**

The retinal pigment epithelium (RPE) is critical in the pathophysiology of retinal diseases, such as age‐related macular degeneration. Adaptive optics transscleral flood illumination (AO‐TFI) offers rapid, detailed morphometric characterization of the RPE layer. This study evaluated AO‐TFI's efficacy and feasibility to detect clinically relevant morphological characteristics in a clinical setting.

**Methods:**

A total of 230 participants, categorized by their central retinal health, underwent comprehensive examination including SD‐OCT, fundus imaging and AO‐TFI using the Cellularis® prototype 2.0. Image quality control and RPE layer quantification were performed with an AO‐TFI‐specific automated segmentation algorithm. Density and area of hyper‐ and hyporeflective regions in the RPE layer plane, and, if detectable, RPE cell density, were quantified. We hypothesized that the RPE cell density would be lower in diseased retinas than in healthy retinas. Imaging results of healthy participants were statistically compared to those of diseased eyes. Additionally, generalized linear and logistic regression mixed‐effect models identified associations between ocular characteristics and imaging parameters.

**Results:**

After quality evaluation, high‐quality images from 200 subjects (87%) were selected and segmented. The number of hyperreflective regions and their mean surface area were significantly higher in diseased than in healthy eyes (68 ± 40/mm^2^ vs. 51 ± 39/mm^2^; *p* < 0.001; 302 ± 196 μm^2^ vs. 155 ± 55 μm^2^; *p* < 0.001). The RPE distinct mosaic pattern was more often visible in healthy retinas (*n* = 103 vs. *n* = 30, *p* < 0.0001). The mean RPE cell density was 6354 ± 695/mm^2^, with comparable counts for healthy and diseased, 6327 ± 687/mm^2^ vs. 6532 ± 725/mm^2^ (*p* = 0.1).

**Conclusion:**

AO‐TFI detected differences between healthy and diseased eyes, indicating its potential as a promising clinical modality providing quantitative and qualitative insights into RPE layer dynamics.

## 1 PURPOSE

The retinal pigment epithelium (RPE) plays a crucial role in maintaining retinal homeostasis by supporting photoreceptor function, regulating the immune response and contributing to choroidal vascular development (Amram et al., 2017; Fuhrmann et al., 2014). Dysfunction of the RPE has been implicated in the pathogenesis of various retinal diseases, including age‐related macular degeneration (AMD) and diabetic retinopathy, as it leads to an imbalance in tissue homeostasis and cellular integrity (Pavan & Dalpiaz, 2018; Simó et al., 2010). Histological and imaging studies have provided insights into RPE alterations in retinal diseases, aiding in the interpretation of high‐resolution retinal imaging techniques (Cuenca et al., 2020). Given the pivotal role of the RPE in retinal health and disease, there is a growing interest in developing imaging modalities that allow for direct in vivo assessment of its morphology and function. Especially, with existing treatments and continued development of novel therapeutics for these retinal disorders (Hoy, 2021; Liao et al., 2020, 2022), there is a need for effective methods to assess and monitor their impact on morphology and function.

Despite significant progress in retinal imaging modalities, including high‐resolution spectral‐domain optical coherence tomography (SD‐OCT) and fundus autofluorescence (AF), direct visualization of the RPE at the cellular level remains challenging due to limitations in axial resolution, contrast mechanisms and optical aberrations (Burns et al., 2019; Morgan et al., 2023; Rossi et al., 2021). While SD‐OCT and AF provide valuable structural and metabolic information about the RPE, they lack the resolution necessary to resolve individual RPE cells in vivo (Sadda et al., 2018; Spaide, 2018). Recent advances in adaptive optics (AO) imaging have improved cellular‐level visualization, yet challenges remain in terms of acquisition time and field of view (Akyol et al., 2021; Liu et al., 2017). Adaptive optics transscleral flood illumination (AO‐TFI) imaging is among these technical advances (Laforest et al., 2020). This technology employs transscleral illumination of the fundus through dual oblique beams, allowing for the acquisition of reflected light from the RPE layer (Govindahari et al., 2024; Laforest et al., 2020).

Over the past two decades, significant progress has been made in the development and implementation of adaptive optics (AO) for high‐resolution in vivo retinal imaging. AO technologies integrated with scanning laser ophthalmoscopy (AO‐SLO) and spectral‐domain optical coherence tomography (SD‐OCT) have enabled direct visualization of individual photoreceptors and retinal microstructures (Morgan et al., 2023). In parallel, multiple groups have sought to overcome the technical challenges of imaging the RPE, particularly the confounding signal from the overlying photoreceptor mosaic. Early efforts using AO‐enhanced autofluorescence demonstrated the feasibility of RPE cell visualization (Granger et al., 2018; Morgan et al., 2009; Tam et al., 2016; Vienola et al., 2020), while AO‐OCT techniques further advanced the capability for three‐dimensional RPE morphometry (Liu et al., 2016; Shirazi et al., 2020).

Despite these achievements, limitations remain—especially regarding fields of view, acquisition time, and the complexity of alignment and interpretation in clinical settings. Recent innovations have sought to address these barriers. Laforest et al. (2020) showed that a transscleral flood illumination technique combined with an AO fundus camera can capture high signal‐to‐noise images of human RPE, photoreceptors and nerve fibre layer using near‐infrared light (*λ* = 850 nm) introduced through the nasal and temporal sclera into the eye by two light‐emitting diodes positioned laterally (Laforest et al., 2020). This approach benefits from increased light penetration to deeper retinal layers and reduced photoreceptor signal dominance, offering a more direct pathway for visualizing the RPE mosaic.

The aim of this study was to build on this methodology to acquire high‐resolution, in vivo images of the human RPE across the central retina and to systematically quantify and compare RPE layer morphometric characteristics in healthy and diseased retinas.

## 2 METHODS

### 2.1 Design

This cross‐sectional clinical study was conducted at the Eye Clinic of the Cantonal Hospital Lucerne between May 2021 and October 2022. The study was conducted in accordance with the Declaration of Helsinki and ISO 14155. The protocol (ClinicalTrials.gov: NCT04912622; kofam.ch, SNCTP000004502) was approved by the regional ethics committee (BASEC: EKNZ2020‐02454) and the Swiss Agency for Therapeutic Products (Swissmedic).

### 2.2 Recruitment

Adults, with a diagnosed retinal disorder undergoing treatment at the Medical Retina Clinic, with no history of epilepsy or pregnancy, and who were able and agreed to adhere to the study protocol were eligible for the study. Participants with healthy retinas were eligible if there was no known history of retinal disease prior to inclusion. The participants for the healthy retina group were recruited in the pre‐cataract examination clinic and from accompanying relatives of patients, and volunteers from the Eye Clinic. Written informed consent was obtained from all participants prior to enrolment.

### 2.3 Ocular examination and image acquisition

Demographics, and ocular characteristics, for example lens status (i.e., presence of cataract), pupil size, and iris colour (categorized into light [blue, green, light brown, grey] and dark [brown, dark green‐brown]), visual acuity, multimodal imaging data, including SD‐OCT (Spectralis®, Heidelberg Engineering, Germany) and adverse events (AEs) during image acquisition, were collected for all participants and all eyes. Prior to AO‐TFI imaging with Cellularis® (prototype version 2.0, EarlySight, Switzerland), axial length (AL) was measured by optical biometry (IOL‐Master 700®, Zeiss, Germany; Optical Biometer OA‐2000, Tomey, Japan). Two trained personnel evaluated the SD‐OCTs and autofluorescence images. AMD was categorized as AMD with geographic atrophy (GA), neovascular AMD or AMD with drusen. AMD with GA was defined as the presence of any type of retinal atrophy (Holz et al., 2017; Sadda et al., 2018). Neovascular AMD was defined based on the presence of typical OCT characterization of choroidal neovascularization (Keane et al., 2012). Eyes presenting with drusen but showing no signs of GA or neovascularization on OCT were defined accordingly (Spaide & Curcio, 2010; Zhang & Sivaprasad, 2021). The localization of AMD‐specific retinal changes was documented (i.e. foveal or extrafoveal). Furthermore, the presence and location of other retinal disorders (e.g., macular holes or epiretinal membranes and SD‐OCT irregularities) were noted.

**FIGURE 1:**
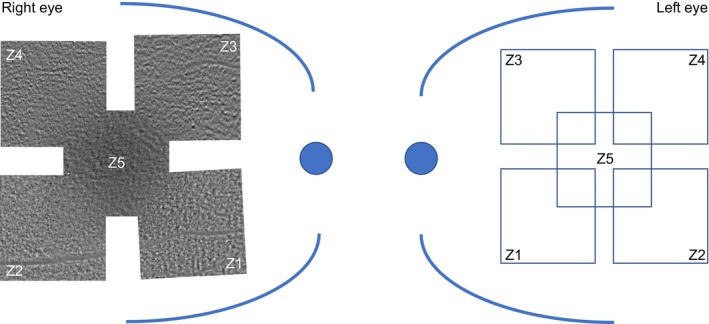
AO‐TFI image acquisition scheme. Images were acquired for five predefined zones (Z1–Z5), with approximate coordinates mirrored to the fovea for the left (LE) and right (RE) eyes. Each zone measured 2 × 2 mm (6.7° × 6.7° field of view) and was sampled with 2048 × 2048 pixels. The compounded arrangement of the five zones formed an X‐shaped area covering a larger region of the central macula. Device digital resolution: 0.8–1.2 μm/pixel; correction for axial length variation was conducted for image quantification.

AO‐TFI imaging was performed using the Cellularis® prototype 2.0 device. Whenever feasible, both eyes were imaged. Five predefined regions of interest of the RPE layer within the central macula, each measuring 2 × 2 mm (6.7° × 6.7° field of view), were captured and positioned in an X‐shaped configuration, resulting in a much larger compounded X‐shaped area (Figure 1). The focal plane was adjusted based on the spherical equivalent refraction specified for each eye at the beginning of image acquisition. Additionally, manual adjustments could be made via the software interface to ensure an optical focal plane. Device digital resolution is 0.8–1.2 μm/pixel, correction for axial length variation was conducted during and for the image quantification process. An internal yellow 580‐nm LED target served as a fixation point for each of the five zones, while an external fixation target was provided for participants with reduced visual acuity and altered central fixation. The mean duration for image acquisition was approximately 1 min, with five zones acquired per eye, resulting in an overall examination duration of 5–6 min per eye. During image acquisition, technical difficulties, subject‐related factors (e.g. poor fixation or eye movements) and some undefined causes occasionally compromised image quality. These acquisition problems were prospectively recorded and categorized as technical, subject‐related or undefined. The frequency of these issues was tracked and analysed across healthy and diseased groups to assess their impact on image quality.

### 2.4 Image analysis and quantification

A proprietary software developed by EarlySight SA was utilized for automatic image quality analysis and segmentation. The software uses a machine learning algorithm specifically trained to assess AO‐TFI images based on the presence of blurring, noise and retinal vessels, the lack of local focus or other imaging artefacts. Based on these factors, the visible image area was automatically assessed for every image. This algorithmically assigned percentage of visible and hence analysable area of an image, with values ranging from 0.0% to 99.0%, was used for image selection. Images with <20% of visible area were excluded from the analysis. The RPE cell count was only analysed in images where RPE mosaic was continuously visible in the previously assessed visible image area. On a separate test set that the algorithm had not seen during training, it was ensured that the automatically assigned quality values by the algorithm follow the subjective evaluations by humans.

The morphometric analysis did not rely on fixed grey value thresholds but involved automated detection and segmentation of hyper‐ and hyporeflective regions using a machine learning algorithm trained on manually annotated image crops. These analyses were performed separately from RPE mosaic analysis. The parameters generated with the automated segmentation include density of hyper‐ and hyporeflective regions (n/mm^2^) and their mean area (μm^2^). For the subset of images, where the mosaic was visible, the percentage of area of visible mosaic over usable image area, that is mosaic over visible area (%), as well as the mean density (n/mm^2^), mean area (μm^2^) and perimeter (μm) were estimated.

To validate the performance of the automatic segmentation algorithm, manual labelling of structures in a subset of 90 image crops was conducted by five independent graders who had been trained by a medical expert. The mean percentage difference between the number of regions detected by the algorithm and those identified through manual grading was found to be 20.7%. In comparison, the mean percentage difference among the five graders was 19.4%.

### 2.5 Statistical analysis

Statistical analyses were primarily descriptive, providing a comprehensive overview of the subjects studied and the eyes imaged using AO‐TFI. We compared image quality metrics between healthy and diseased retinas, as well as the characteristics of subjects and eyes with segmented images. Additionally, we summarized the segmentation results and RPE characteristics for participants with healthy versus diseased retinas. Group comparisons were conducted using the Wilcoxon signed‐rank sum test (per independent) and the Student's *t*‐test for numeric, and chi‐squared for categorical data. Two‐sided ANOVA was used to compare means if more than two groups were compared. For questions about association, for example investigating ocular characteristics associated with poorer image quality, that is smaller percentage of visible and analysable image, linear mixed‐effect models considering participants eye as random effects were conducted. Similarly, univariate and multivariable logistic mixed‐effect models were applied to investigate the association of ocular and imaging parameters with diseased retina. A secondary analysis was performed on an age‐matched study participants' subset. *p* values <0.05 were considered significant. Statistical analyses were performed using R version 4.1.0 (2021‐05‐18).

## 3 RESULTS

### 3.1 AO‐TFI image acquisition and quality

**FIGURE 2:**
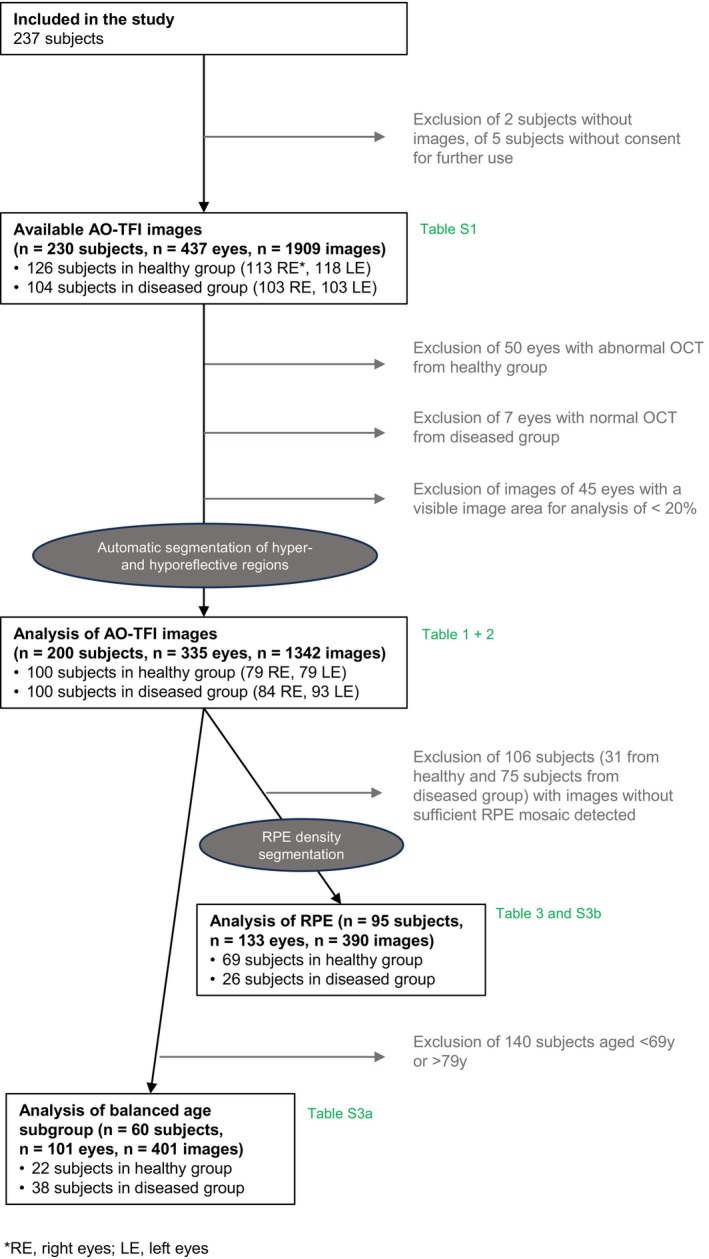
Study flow chart describing eye and image inclusion and selection for analysis. (The tables reporting the details of the analysed eye and the images are noted in green.)

Of 237 participants recruited into the study, 230 had at least one zone from one of their eyes imaged with Cellularis® Prototype 2.0, leading to a total of 1909 images of zones from 437 eyes (Figure 2). Comparison of characteristics of study participants (*n* = 230) and their eyes (*n* = 437), as well as frequency of image acquisition problems, number of captured images and frequency of adverse events are summarized in Table S1. Image acquisition problems were more frequently reported in the diseased retina group (50% vs. 35%; *p* = 0.002), with most difficulties being participant‐related (e.g. inability of target fixation or head movement during image acquisition). Adverse events reported during or after image acquisition were reported by 6% of participants and included minor events. The mild adverse events were reported with similar frequency in both healthy and diseased groups. These events, primarily dry eyes and mild headaches or neck discomfort related to the imaging procedure, were transient and resolved without intervention when inquired during routine follow‐up calls, one week post examination.

**FIGURE 3:**
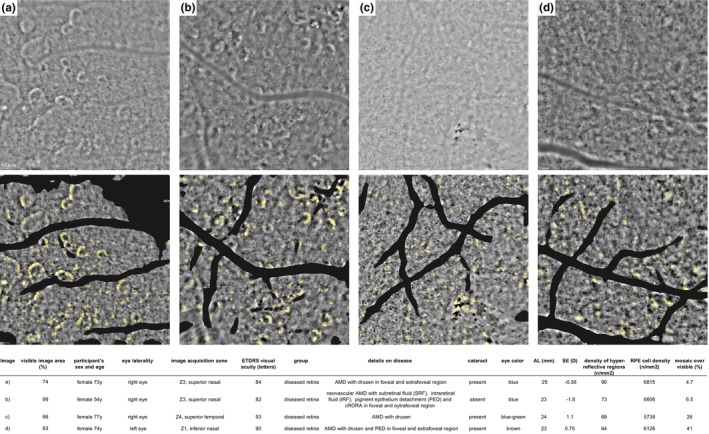
Example AO‐TFI images from four diseased eyes (a–d). Each panel shows the same image twice: The top row displays the raw AO‐TFI image; the bottom row shows the identical image zone with a black mask indicating unusable areas (e.g. retinal vessels, low signal‐to‐noise) excluded from segmentation and quantitative analysis. The four cases illustrate varying percentages of visible RPE mosaic (74% to 86%); image and eye characteristics are summarized in the accompanying table.

AO‐TFI images with at least 20% visible image area were included for analysis and quantification of morphometric characteristics of the RPE layer. When multiple images of the same zones and eyes were available, the one with the larger percentage of visible image area was included. Four AO‐TFI images of diseased eyes, along with their image details, including percentage of visible image area and ocular characteristics are depicted in Figure 3.

After selecting images with at least 20% visible image area, a total of 1342 images acquired from 335 eyes (healthy retina: 662 images acquired from 158 eyes of 100 subjects; diseased retina group: 680 images acquired from 177 eyes of 100 subjects) were analysed, segmented for hyper‐ and hyporeflective regions quantification, and further evaluated whether suitable for the quantification of the RPE cells.

Information and comparison of the 200 participants and their 335 eyes, of which images could be quantified, are summarized in Table 1. Image acquisition problems were present in 38% of all cases. Specifically, 9% were due to technical issues, 23% to subject‐related factors and 6% remained undefined. The healthy group showed fewer acquisition problems (27%) compared to the diseased group (47%) (*p* < 0.001). Technical problems accounted for 11% in the healthy group and 7% in the diseased group, while subject‐related issues were more frequent in the diseased group (39%) than in healthy subjects (4%).

**TABLE 1:** Characteristics of study participants (*n* = 200) and eyes (*n* = 335) with analysed AO‐TFI images (*n* = 1342).

	Overall cohort	Healthy retina	Diseased retina	Statistics
Subjects (*n*)	200	100	100	
Female	115 (57%)	55 (55%)	60 (60%)	*p* = 0.6, *X* ^2^ = 0.3, df = 1
Male	85 (43%)	45 (45%)	40 (40%)	
Age (years)				
Mean ± SD	65 ± 19	54 ± 19	76 ± 11	*p* < 0.001, *W* = 1139
Median	72	60	78	
Min: max	23: 94	23: 86	31: 94	
Eyes (*n*)	335	158	177	
Right	163 (49%)	79 (50%)	84 (47%)	*p* = 0.7, *X* ^2^ = 0.1, df = 1
Left	172 (51%)	79 (50%)	93 (53%)	
Visual acuity ETDRS				
Mean ± SD	81 ± 11	86 ± 6	76 ± 13	*p* < 0.001, *W* = 21 913
Median	84	88	80	
Min: max	15: 95	63: 95	15: 95	
Spherical equivalent (D)				
Mean ± SD	−0.2 ± 2.0	−0.8 ± 2.2	0.3 ± 1.5	*p* < 0.001, *W* = 10 700
Median	0.0	−0.1	0.1	
Min: max	−7.9: 4.6	−7.9: 3.2	−4.4: 4.6	
Axial length (mm)				
Mean ± SD	23.5 ± 1.2	23.8 ± 1.2	23.3 ± 1.1	*p* < 0.001, *W* = 17 690
Median	23.4	23.6	23.2	
Min: max	20.7: 27.7	21.9: 27.7	20.7: 26.7	
Astigmatism >1.5D (*n*)				
Absent	281 (84%)	146 (92%)	135 (76%)	*p* < 0.001, *X* ^2^ = 15, df = 1
Present	54 (16%)	12 (8%)	42 (24%)	
Pupil size (*n*)				
Small <3 mm	56 (17%)	39 (25%)	17 (9%)	*p* < 0.001, *X* ^2^ = 66, df = 3
Medium 3–5 mm	228 (68%)	74 (46%)	154 (87%)	
Large >5 mm	48 (14%)	43 (27%)	5 (3%)	
NA	3 (1%)	2 (2%)	1 (1%)	
Eye colour (*n*)				
Light	218 (65%)	86 (54%)	132 (74%)	*p* < 0.001, *X* ^2^ = 15, df = 2
Dark	114 (34%)	70 (44%)	44 (25%)	
NA	3 (1%)	2 (2%)	1 (1%)	
Image acquisition problems (*n*)				
Absent	209 (62%)	116 (73%)	93 (53%)	*p* < 0.001, *X* ^2^ = 15, df = 1
Present	126 (38%)	42 (27%)	84 (47%)	
Technical	29 (9%)	17 (11%)	12 (7%)	
Subject‐related	76 (23%)	6 (4%)	70 (39%)	
Undefined	21 (6%)	19 (12%)	2 (1%)	
Cataract (*n*)				
Absent	197 (59%)	90 (57%)	107 (60%)	*p* = 0.6, *X* ^2^ = 0.3, df = 1
Present	138 (40%)	68 (43%)	70 (40%)	
Posterior capsular cataract	23 (7%)	16 (10%)	7 (4%)	
Retinal disease (*n*)				
None	158 (47%)	158 (100%)	0 (0%)	*p* < 0.001, *X* ^2^ = 328, df = 1
Present	177 (53%)	0 (0%)	177 (100%)	
Neovascular AMD	72 (21%)		72 (41%)	
Drusen or GA+	92 (27%)		92 (52%)	
Other	13 (5%)		13 (7%)	

*Note*: Wilcoxon nonparametric test was used for numeric variables, Chi‐squared test for categorical data.

Abbreviations: + GA, geographic atrophy; AMD, age‐related macular degeneration.

Among the five different acquisition zones (z1–z5, as illustrated in Figure 1), a similar proportion of images of all five zones were analysed of the two groups (*p* = 1.0; Table 2). This suggests that all zones could be imaged successfully to a comparable extent across both groups, and across all imaged retinal regions. Despite the exclusion of low‐quality images (<20% visible image area), the mean visible image area in percentage remained significantly lower in the diseased retina group than in the healthy group (82 ± 13% vs. 62 ± 22%; *p* < 0.001; Table 2).

**TABLE 2:** Results of image segmentation analysis of 200 subjects, their 335 eyes and acquired AO‐TFI images (*n* = 1342).

	Overall cohort	Healthy retina	Diseased retina	Statistics
Eyes (*n*)	335	158	177	
Right	163 (49%)	79 (50%)	84 (47%)	*p* = 0.7, *X* ^2^ = 0.1, df = 1
Left	172 (51%)	79 (50%)	93 (53%)	
Zones with images (*n*) with ≥20% visible area for analysis	1342	662	680	
*z*1	292	147	145	*p* = 1.0, *X* ^2^ = 0.2, df = 4
*z*2	265	130	135	
*z*3	264	129	135	
*z*4	272	133	139	
*z*5	249	123	126	
Visible image area %				
Mean ± SD	72 ± 21	82 ± 13	62 ± 22	*p* < 0.001, *W* = 400 000
Median	80	86	63	
Min: max	20: 99	22: 99	20: 98	
Density of hyperreflective regions (n/mm^2^)				
Mean ± SD	60 ± 41	51 ± 39	68 ± 40	*p* < 0.001, *W* = 200 000
Median	59	50	68	
Min: max	0: 182	0: 153	1: 182	
Mean area of hyperreflective regions (μm^2^)				
Mean ± SD	230 ± 162	155 ± 55	302 ± 196	*p* < 0.001, *W* = 71 703
Median	192	153	256	
Min: max	42: 2659	42: 390	49: 2659	
Density of hyporeflective regions (n/mm^2^)				
Mean ± SD	80 ± 42	78 ± 45	83 ± 38	*p* = 0.06, *W* = 200 000
Median	81	80	81	
Min: max	1: 223	1: 184	4: 223	
Mean area of hyporeflective regions (μm^2^)				
Mean ± SD	222 ± 125	155 ± 54	259 ± 287	*p* < 0.001, *W* = 70 998
Median	189	151	140	
Min: max	50: 1057	50: 384	72: 1057	

*Note*: Wilcoxon nonparametric test was used for numeric variables, Chi‐squared test for categorical data.

### 3.2 Participant demographics and eye characteristics

As mentioned before, demographics and clinical characteristics of the participants (*n* = 200) and their eyes (*n* = 335) selected for segmentation are summarized in Table 1. Sex distribution was well‐balanced between the groups (healthy retina: 55% female; diseased retina: 60% female) but age was significantly higher in the diseased retina group versus the healthy retina group (76 ± 11 vs. 54 ± 19 years; *p* < 0.001). As expected, visual acuity was significantly lower in the diseased retina group (*p* < 0.001). Also, significantly longer, that is more myopic eyes were found in the healthy group, as compared to the diseased retina group (*p* < 0.001). The pupil size was on average larger in the healthy retina group (*p* < 0.001), since the participants were recruited from the pre‐cataract consultation, where they had their pupils dilated. Cataracts were similarly often found in both groups (*p* = 0.6). Interestingly, the proportion of light‐coloured eyes was significantly higher in the diseased group (74% vs. 54%, *p* = 0.001). In the diseased retina group, 93% of the eyes were graded as affected by AMD (drusen or dry AMD in 41%; neovascular AMD in 52%), in the remaining seven percent various abnormalities of the central retina, including epiretinal membranes were observed.

### 3.3 Comparison of RPE layer characteristics in healthy retina vs. diseased retina participants

**FIGURE 4:**
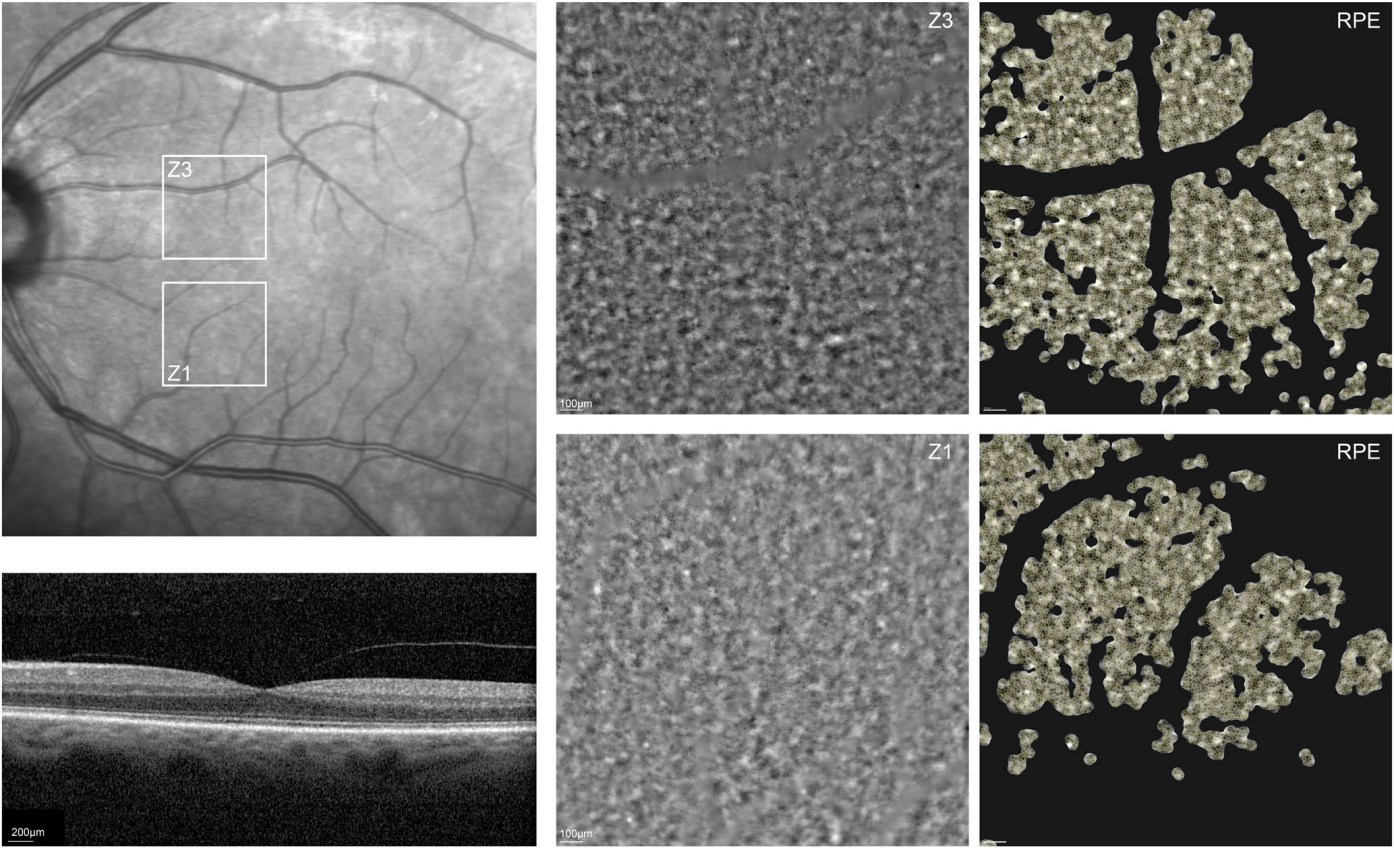
AO‐TFI image zones (Z1 and Z3) of a healthy left eye of a 70‐year‐old male patient, outlined and superimposed on a near‐infrared en‐face fundus image. Corresponding central horizontal OCT B‐scan through the fovea is shown for illustration (lower left). Larger scale images of Z1 and Z3 are displayed on the right, along with their corresponding RPE segmentation results (segmentation lines are marked in yellow). Characteristics of the imaged eye: 80 letters in the ETDRS visual acuity test; healthy eye; cataract; dark (brown) iris colour; axial length of 22 mm; spherical equivalent refraction (SE) −3.1 diopters (D); estimated density of hyperreflective regions 105/mm^2^; RPE cell density 5884/mm^2^; mosaic over visible 38%.

**FIGURE 5:**
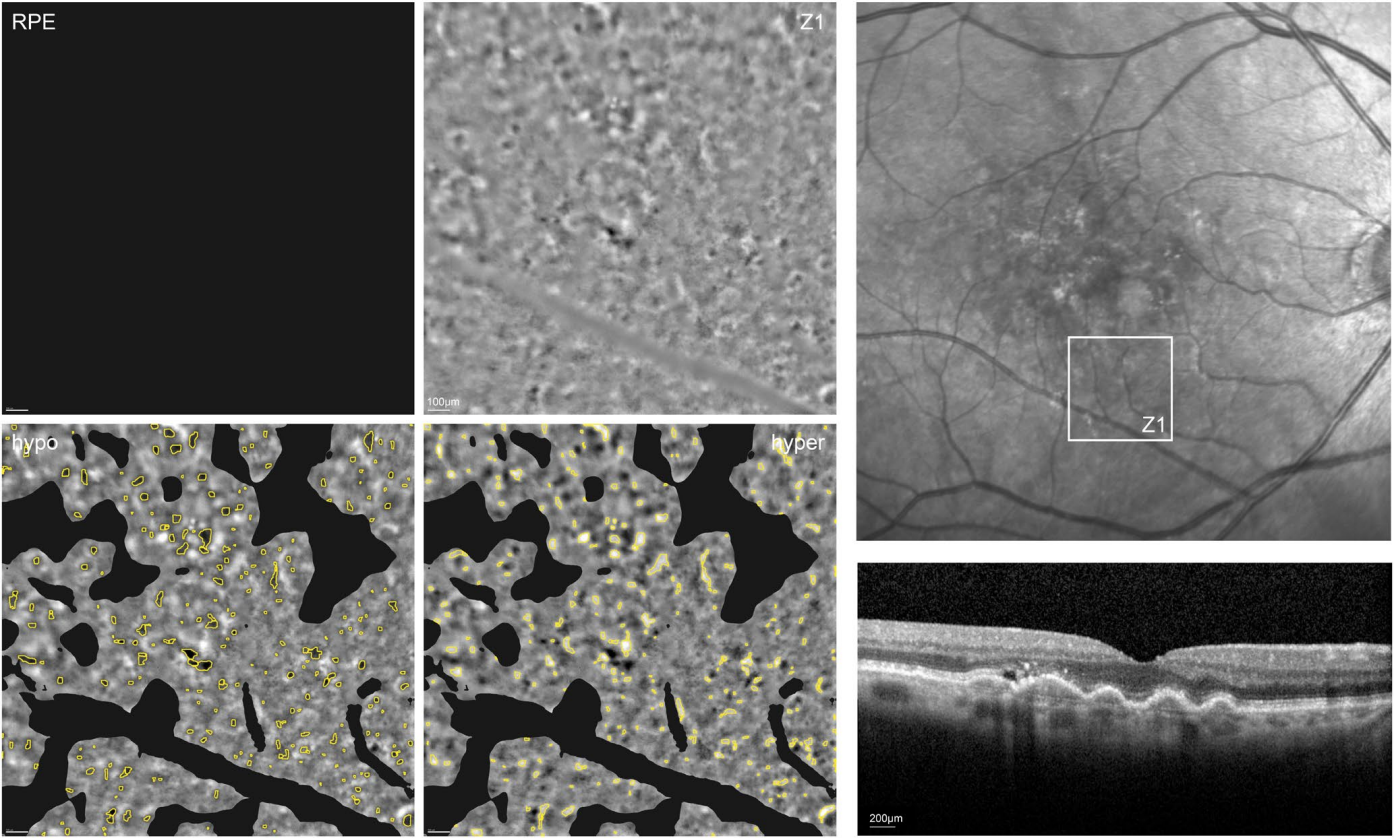
AO‐TFI image of zone Z1 (inferonasal) of a diseased right eye (female, 76 years), with a visible image area of 71%, outlined and superimposed on a near‐infrared en‐face fundus image. A central horizontal OCT B‐scan through the fovea is shown for illustration of disease state (lower right). A larger scale AO‐TFI image of Z1 is displayed on the left, along with its corresponding RPE layer segmentation results. In this case, the RPE could not be quantified, as there was not enough visible mosaic within the imaged area. The lower row depicts the two segmentation overview images for hypo‐ and hyperreflective regions, respectively (segmentation lines are marked in yellow). Characteristics of the imaged eye: 90 letters in the ETDRS visual acuity test; AMD with drusen in foveal and extrafoveal regions; cataract; brown iris colour; axial length of 23 mm; SE −0.38 D; estimated density of hyperreflective regions 105/mm^2^; RPE cell density NA; mosaic over visible 0%.

Examples of AO‐TFI images, along with corresponding segmentation for both healthy and diseased eyes, are presented in Figure 4 (healthy) and Figure 5 (diseased). In Figure 4, AO‐TFI images (zone Z1, and Z3) of a left eye of a 70‐year‐old male patient is superimposed on a near‐infrared en‐face fundus image, along with a corresponding central horizontal OCT b‐scan on the fovea acquired with Heidelberger Spectralis®. Larger scale of the images of Zones 1 and 3 are displayed on the side along with their corresponding RPE segmentation results. The imaged eye had a brown iris (i.e. dark colour), measured an axial length of 22 mm, with a spherical equivalent refraction (SE) of −3.1 dioptres (D), for the image Zone 1 an estimated density of hyperreflective regions 105/mm^2^ (segmentation not shown in Figure 4), as well as a RPE cell density of 5884/mm^2^ with a mosaic visible area over visible image area of 38% were determined. Similar values also for the image of Zone 3 were detected. In the segmentation images, the yellow lines delineate the contours of the segmented regions. The regular mosaic pattern of RPE cells is easily discernible in the healthy example depicted in Figure 4. Conversely, in the diseased retina shown in Figure 5, the mosaic pattern is markedly less distinguishable. In the diseased case, the presence of other hypo‐ and hyperreflective structures is observable, highlighting the alterations in RPE cell organization associated with retinal pathology. The displayed AO‐TFI image was captured for Zone 1 (Z1; inferonasal to fovea) in a diseased right eye of a 76‐year‐old female patient, the visible image area of the sample image was assed to be 71%. In the central horizontal OCT b scan on the fovea acquired with Heidelberger Spectralis® alterations of the RPE layer with pigment epithelium detachment (PED) and drusen related to age‐related macular degeneration can be seen. The patient reached a good visual acuity of 90 letters in the ETDRS visual acuity test on the right, she has brown eyes, SE of −0.38 D, and the axial length measured 23 mm. The enlarged AO‐TFI image of Zone 1 is displayed on the left of the montage, along with segmentation results. It can be appreciated that in this case, the RPE could not be quantified, as there was not enough visible mosaic over the visible image area. The number of hyper‐ and hyporeflective regions, however, could be analysed (e.g. estimated density of hyperreflective regions 105/mm^2^).

In Table 2, the results of the image quantification of 1342 images are reported: The mean density of hyperreflective regions was 60 ± 41/mm^2^ overall, and it was significantly lower in the healthy group (51 ± 39/mm^2^ vs. 68 ± 40/mm^2^; *p* < 0.001). Also, the mean area of the hyperreflective regions was found to be significantly smaller (155 ± 55 μm^2^ vs. 302 ± 196 μm^2^; *p* < 0.001) in the eyes belonging to the healthy group compared with the diseased group. Similarly, there was a difference in the number and especially the size of the hyporeflective regions.

In the study flow chart (Figure 2), it is marked that only in 390 images of 95 subjects and 133 eyes, the RPE mosaic was sufficiently visible over the visible image area, to be quantified. Results of the RPE segmentation are summarized in Table 3. Finally, the mean RPE cell density was estimated to be 6327 ± 687/mm^2^ in the healthy group and 6532 ± 725/mm^2^ in the diseased group (*p* = 0.001). The boxplots in Figure S1 illustrate the comparison of RPE layer segmentation characteristics, that is density of hyper‐ and hyporeflective regions, as well as RPE density. No difference was found in mean RPE cell density for the five different zones of image capture (*p* = 0.68, s. boxplots in Figure S2). An additional analysis in an age‐balanced subgroup (>69 and <79 years) was conducted and did not reveal any new findings (see Table S3a,b).

**TABLE 3:** Results of RPE segmentation analysis in images with visible RPE mosaic (*n* = 390) of 95 subjects and their 133 eyes.

	Overall cohort	Healthy retina	Diseased retina	Statistics
Subjects (*n*)	95	69	26	
Female	55 (58%)	38 (55%)	17 (65%)	*p* = 0.5, *X* ^2^ = 0.5, df = 1
Male	40 (42%)	31 (45%)	9 (35%)	
Age (years)				
Mean ± SD	56 ± 19	51 ± 19	68 ± 19	*p* < 0.001, *W* = 400
Median	62	54	74	
Min: max	23: 86	23: 79	31: 86	
Eyes (*n*)	133	103	30	
Right	67 (50%)	52 (50%)	15 (50%)	*p* < 0.001, *X* ^2^ = 79.15, df = 1
Left	66 (50%)	51 (50%)	15 (50%)	*p* = 1.0, *X* ^2^ = 0, df = 1
Zones with images (*n*) with ≥20% visible area for analysis	390	339	51	
*z*1	81	73	8	*p* = 0.1, *X* ^2^ = 8, df = 4
*z*2	70	63	7	
*z*3	100	80	20	
*z*4	80	68	12	
*z*5	59	55	4	
Mosaic over visible area %				
Mean ± SD	29 ± 21	30 ± 21	16 ± 16	*p* < 0.001, *W* = 11 963
Median	28	31	10	
Min: max	0: 64	0: 64	0: 52	
Density of RPE (n/mm^2^)				
Mean ± SD	6354 ± 695	6327 ± 687	6532 ± 725	*p* = 0.1, *W* = 7451
Median	6291	6279	6456	
Min: max	4605: 8476	4605: 8476	5269: 8458	
Mean cell area raw (μm^2^)				
Mean ± SD	162 ± 18	162 ± 18	158 ± 16	*p* = 0.3, *W* = 9500
Median	161	161	158	
Min: max	119: 220	119: 220	120: 191	
Mean cell perimeter (μm)				
Mean ± SD	50 ± 2.7	50 ± 2.7	50 ± 2.5	*p* = 0.3, *W* = 9420
Median	50	50	50	
Min: max	43: 59	43: 59	43: 54	

*Note*: Wilcoxon nonparametric test was used for numeric variables, Chi‐squared test for categorical data.

**FIGURE 6:**
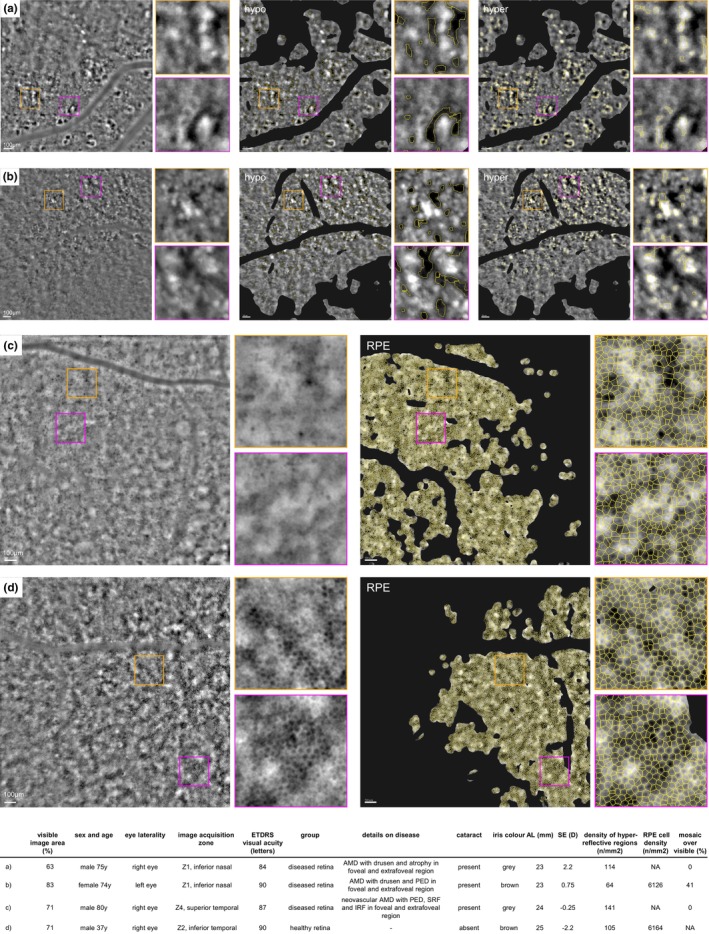
(a–d) With accompanying table. Examples of AO‐TFI images of three diseased (*n* = 3) and healthy (*n* = 1) retinas, including their corresponding segmentation results for hypo‐ and hyperreflective regions quantification (a, b). Original AO‐TFI images on the left with magnified crops in orange and pink on the right. Corresponding segmentation results for hyper‐ and hyporeflective regions. Examples of RPE segmentation results are shown in c, d. Characteristics of selected eyes in the accompanying table.

Examples of images and their segmentation, acquired from three diseased retinas and one healthy retina are shown in Figure 6a–d. Additional examples from a series of diseased eyes are presented in Figure S3a–h, along with the detailed description.

#### 3.3.1 Association of ocular factors with image quality and disease state

To understand the factors influencing AO‐TFI image quality, we analysed subject characteristics in relation to the visible image area. Table S2 highlights these associations, revealing that increasing age and the presence of astigmatism of −1.5D or more significantly decreased the visible area (*p* < 0.001). Light eye colour, image acquisition problems and retinal disease were also associated with reduced image quality (*p* < 0.001). Conversely, axial length positively influenced visible area (*p* = 0.004), while sex, cataract status and pupil size showed no significant effects.

Furthermore, we used univariate and multivariable logistic mixed‐effect models to investigate the association of both subjects' and ocular characteristics, as well as AO‐TFI segmentation results with retinal disease status (Table 4). Univariate analysis revealed that older age and shorter axial length were significantly associated with the presence of retinal disease (*p* < 0.001). Light eye colour also showed a positive association (*p* < 0.001). While the density of hyperreflective regions was not significantly linked, the mean area of hyperreflective regions was significantly larger in diseased eyes (*p* = 0.016). Conversely, the mean area of hyporeflective regions showed a strong association with retinal disease (*p* < 0.001). Multivariable models confirmed age, axial length, and eye colour as significant predictors, with older age and light eye colour increasing the likelihood of disease, whereas longer axial length was associated with a decreased risk.

**TABLE 4:** Associations of subjects' characteristics, as well as AO‐TFI segmentation parameters with retinal disease state (*n* = 335 eyes, *n* = 1342 images).

	Retina diseased (y/n)		
	Estimate	SE	Pr (>|*z*|)
Univariate models^a^			
Age	52.47	5.99	<0.001
Sex	0.123	1.036	0.91
Axial length	−16.72	2.69	<0.001
Eye colour			
Light colour	25.35	1.35	<0.001
Hyperreflective regions density	0.461	0.446	0.30
Mean area of hyperreflective regions	2.696	1.119	0.016
Hyporeflective regions density	0.320	0.001	0.61
Mean area of hyporeflective regions	11.52	1.981	<0.001
Multivariable model^b^			
Age	77.59	10.02	<0.001
Axial length	−5.51	1.81	0.002
Eye colour			
Light colour	20.88	4.44	<0.001
Mean area of hyperreflective regions	5.81	3.19	0.068
Mean area of hyporeflective regions	5.03	3.59	0.161

*Note*: For the multivariable model, all parameters were scaled before analysis.

^a^ Logistic regression, using the values of all predictors for a binary outcome.

^b^ No interactions included.

## 4 DISCUSSION

This study presents novel findings using AO‐TFI imaging to characterize RPE layer morphology in a large cohort, distinguishing features between healthy and diseased retinas. Using AO‐TFI imaging, we were able to elucidate in vivo differences in RPE layer morphology, between healthy and diseased retina participants, most of whom had AMD. RPE layer morphometric characteristics, which were automatically quantified included the density of hyper‐ and hyporeflective regions, and their area, as well as‐ for a smaller subset of cases—RPE cell density.

Since AO‐TFI is a relatively new technique that has only recently become available in clinic, there are currently only a few comparable published studies on this imaging technology and its application in healthy and pathologies of the RPE (Gofas‐Salas et al., 2024; Govindahari et al., 2024; Kowalczuk et al., 2025; Vienola et al., 2022). The effectiveness of AO‐TFI imaging shown in our study aligns with previous studies highlighting its advantages over traditional imaging modalities. For instance, investigations into RPE abnormalities in patients with central serous chorioretinopathy (CSCR), demonstrated that AO‐TFI imaging can reveal significant alterations that standard multimodal imaging might overlook, such as detailed hyperreflective structures (Govindahari et al., 2024). These findings suggest that the enhanced cellular resolution provided by AO‐TFI imaging may allow for more accurate assessments of the RPE, particularly in detecting early signs of retinal pathologies (Gofas‐Salas et al., 2024; Govindahari et al., 2024). In our analysis, we identified significant differences in the number and size of hyper‐ and hyporeflective regions in the RPE layer between healthy individuals and those primarily affected by AMD. However, we did not find a statistically significant difference in the RPE cell density between healthy and diseased retinas, which is contrary to previous reports highlighting a decline in RPE cell number with aging and disease progression (Ach et al., 2014; Delori et al., 2011; Ding et al., 2011; Harman et al., 1997; Schmidt & Peisch, 1986). One possible explanation for this apparent discrepancy is that our quantification was limited to areas in which the RPE mosaic could be clearly visualized. In diseased eyes, the fraction of mosaic over visible area was substantially smaller (on average ~16%) than in healthy eyes (~30%). This may inherently restrict the analysis to the ‘better preserved’ regions of the diseased retina, which may bias the density estimates towards values comparable to healthy controls. In other words, where we cannot visualize the RPE cells, we cannot measure them, and thus, areas of true cell loss may not be captured in the current analysis.

Reported values for RPE density also vary considerably between studies, ranging from approximately 3000–4000 cells/mm^2^ in histological analyses (Jonas et al., 2017; Panda‐jonas et al., 1996) to 4500–6000 cells/mm^2^ in in vivo studies using adaptive optics techniques (Granger et al., 2018; Kowalczuk et al., 2022). In our cohort, the mean RPE density was 6354 ± 695 cells/mm^2^ (median: 6291; range: 4605–8476), which lies at the upper end of previously reported in vivo estimates. These discrepancies likely reflect differences in methodology, including cell quantification, retinal eccentricity of the imaged regions and sample size. Although our study did not demonstrate a reduction in mean RPE cell density between healthy and diseased eyes, the comparatively larger size of our cohort, relative to most previous AO‐based reports, strengthens the robustness of our estimates.

The ability of AO‐TFI imaging to visualize these distinctions in hyper‐ and hyporeflective patterns reinforces its potential role in both clinical diagnostics and research applications. In this sense, the CSCR study reported RPE alterations in regions considered healthy by conventional imaging (Govindahari et al., 2024). Similarly, we can find both hyper‐ and hyporeflective structures also in eyes, which were clinically assessed to be healthy and did not show any OCT abnormalities. However, the density and especially the size of both hyper‐ and hyporeflective regions were associated with disease. This holds true, also when including other factors such as age and axial length into the statistical model.

Qualitatively and quantitatively, we observed a difference in the morphological RPE layer, including a loss of the typical RPE mosaic pattern and an increase of regions with larger hyper‐ and hyporeflectiveness, which may reflect underlying cytoskeletal and functional changes associated with the mechanisms involved in disease progression. Apart from Govindahari et al. (2024) and Kowalczuk et al. (2025) using AO‐TFI, other groups utilizing other AO‐techniques, such as AO‐SLO have identified alterations in hyperreflective structures (Vienola et al., 2022; Vogel et al., 2017). Potentially, such alterations in RPE layer structure could significantly impact the RPE cell's barrier properties and potentially contribute to the pathogenesis of retinal diseases, including AMD. This observation aligns with previous research that links RPE changes to degenerative processes in related conditions (Ach et al., 2014; Delori et al., 2011; Ding et al., 2011; Harman et al., 1997; Schmidt & Peisch, 1986).

Our findings expand this understanding by quantitatively assessing RPE layer characteristics, revealing potential early indicators of disease‐related changes that warrant further investigation. In healthy retinas presenting a typical RPE mosaic, individual cells were effectively identified and quantified. Conversely, in diseased retinas, additional hypo‐ and hyperreflective regions were detected. In all images in which at least 20% of the image area was visible (image selection criterion), the hyper‐ and hyporeflective regions could be detected and quantified. Of these images, RPE mosaic was only visible and hence quantified in just under 30%, most of which belonged to the healthy group. The limited visibility of the RPE mosaic even in healthy eyes may reflect several factors: the RPE layer may not always lie precisely in the optimal focal plane of AO‐TFI; inter‐individual differences in pigmentation and melanin content may influence image contrast; and residual optical artefacts, light scattering, or overlying structures may obscure the signal. In diseased eyes, additional factors such as structural disruption of the outer retina, local atrophy, or deposition of subretinal material are likely to further impair mosaic visibility.

In the 390 images of 95 eyes that could be used for RPE quantification, the RPE density was estimated to be 6354 *±* 695/mm^2^. Our estimated mean RPE cell counts/mm^2^ were similar to previously reported values in vivo studies (Granger et al., 2018), which reported a range of approximately 4500–6000/mm^2^. Previous studies by Rossi et al. (2021) and Vienola et al. (2020) utilized autofluorescence imaging to visualize the RPE mosaic in AMD but did not report quantitative metrics such as the number of hyper‐ and hyporeflective regions or RPE cell density (Rossi et al., 2021; Vienola et al., 2020). Our study is distinguished by providing quantitative morphometric analysis over a larger cohort, including both healthy and diseased eyes, thus attempting to complement prior descriptive work. Previous histological studies, reported lower numbers of RPE cells in the posterior pole (approximately 3000/mm^2^) (Jonas et al., 2017). Our mean cell count of 6327 ± 687/mm^2^ for healthy retinas was also similar, or slightly higher than that of the other published study comparing AO‐TFI with autofluorescence adaptive optics scanning laser ophthalmoscopy; however, their observations were based on only four healthy subjects (Gofas‐Salas et al., 2024). Also, Kowalczuk et al. reported mean RPE cell counts in the range of 3500–4000 cells/mm^2^ using AO‐TFI, they too had a smaller subject sample (Kowalczuk et al., 2022). Differences of RPE cell counts in several studies may be attributable to variations in methodology regarding the imaged regions, as well as demographics of the study participants, including axial length. Overall, data on the regional differences in RPE cell density are still limited. Although it has been reported that the cell number/mm^2^ increases threefold from periphery to fovea (Panda‐jonas et al., 1996), good comparative data from larger populations are not available. We investigate whether there was an association between age and RPE cell density, but despite the wide age range (23 to 86 years), we did not find a statistically significant association between RPE cell density and age in our subgroup analysis of 95 subjects (Table 3, Estimate: 2.50, SE: 2.85, *p* = 0.38). Furthermore, we checked whether there were differences in mean RPE cell density depending on the image zone (Z1–Z4 vs. Z5) and could not find any significant difference in the analysis of variance.

Also, we did not find any methodologically comparable studies measuring the RPE cell count of patients with AMD other than the two of Vienola et al. (2020) and Rossi et al. (2013). It is thought that the number of cells is reduced in patients with AMD but knowledge regarding the extent of the reduction is currently lacking (Bailey‐Steinitz et al., 2020; Holz et al., 2015; Zhang et al., 2023).

Possibly, the hyper‐ and hyporeflective regions identified by AO‐TFI correspond to pathological changes in the RPE layer in diseased eyes, potentially offering enhanced sensitivity to morphological disruptions. Our results here are in line with recently published work by Kowalczuk et al. (2025), which described hyper‐ and hyporeflective alterations such as drusen in OCT with hyporeflective centers and bright edges, crystalline deposits and hyporeflective dots in atrophic regions in eyes with dry AMD using imaged by an earlier AO‐TFI prototype (Kowalczuk et al., 2025). While our primary focus was on comparing healthy and diseased retinas, it is important to note that our diseased group encompassed different stages of AMD, including neovascular (41%) and dry AMD (51%) cases. Most interestingly, we observed such hyper‐ and hyporeflective regions—albeit fewer in number and smaller in size—even in cases where the retina appeared normal on OCT and fundoscopy. In a separate, qualitative analysis of the diseased retina group, we categorized these structures into four different reoccurring patterns, which we were able to correlate with OCT findings, such as different forms of drusen and atrophy. This analysis is part of a separate project, preliminary findings, suggest that AO‐TFI can provide valuable morphological insights, capturing subtle changes associated with AMD progression that may be missed by conventional imaging methods. AO‐TFI thus holds promise for advancing our understanding of how structural and functional alterations in RPE layer relate to AMD progression, thereby offering insights for the development of novel therapeutic strategies.

This study is not without limitations. The inherent variability in image quality, particularly in patients with advanced AMD, may have affected the consistency of the data in the diseased retina group. Furthermore, the age difference between healthy and diseased retina participants was another potential limitation. Increasing age was found to be associated with worse image quality (lower visible and analysable image area). Similar, the age‐adjusted subgroup analysis (*n* = 401 images) showed results that were consistent with the primary analysis (*n* = 1342 images), reinforcing a true and measurable difference in RPE layer morphology, that is density and size of hyper‐ and hyporeflective regions between healthy and diseased retinas. Also, age was not associated with the number or RPE cell density (*n* = 390 images of 95 subjects).

A strength of this study is the large sample size compared with previous investigations. To the best of our knowledge, this is the first study to assess human RPE cell density in both healthy and diseased retinas in a large‐scale clinical setting with AO‐TFI; almost 2000 images were acquired in a systematic manner, following a meticulous image protocol. The automatic segmentation and quantification of data, combined with a larger field of view compared with similar studies (Gofas‐Salas et al., 2024), enabled a comprehensive evaluation of RPE layer morphology.

Various optical imaging techniques, including AO‐SLO with offset aperture, split detector, darkfield, autofluorescence (Granger et al., 2018; Morgan et al., 2009; Tam et al., 2016; Vienola et al., 2020) and AO‐OCT (Liu et al., 2016; Shirazi et al., 2020), have been developed for observing PR and RPE. However, many of these methods, including the one applied by Gofas‐Salas et al., are limited by a small field of view, reduced image clarity or prolonged acquisition times that hinder clinical use (Gofas‐Salas et al., 2024). In contrast, AO‐TFI offers a larger field of view and faster acquisition times, making it well‐suited for clinical applications. The technique enhances the signal‐to‐noise ratio of retinal structures through transscleral flood illumination compared to transpupillary illumination (Laforest et al., 2020). While AO‐SLO and AO‐OCT can provide high‐resolution imaging, their clinical utility is often limited by weak autofluorescence signal strength in AO‐SLO—requiring longer acquisition times—and by the need for registering hundreds of volumes in AO‐OCT to generate intracellular motion contrast (Morgan et al., 2023).

Considering our findings, we conclude that a detailed investigation into RPE layer morphometric patterns in AMD is warranted, potentially paving the way for new biomarkers for early detection and prognostic assessments. Additionally, further comparative studies employing other established imaging modalities could elucidate the interplay between RPE and associated retinal structures, thereby enhancing our understanding of retinal health and disease dynamics. Future research should focus on longitudinal studies of RPE characteristics in AMD cases, employing AO‐TFI imaging to monitor disease progression.

In conclusion, our study confirms that AO‐TFI effectively detected specific differences of the RPE layer between healthy and diseased eyes, indicating its potential as a promising clinical modality in retinal disorders, such as AMD. The results display the utility of AO‐TFI imaging as a tool for investigating RPE dynamics in both health and disease, emphasizing its ability to discern subtle morphological changes. Continued exploration of the role of RPE within the broader context of retinal pathology remains essential for improving patient outcomes in ophthalmology. Its application in the management of AMD and other retinal disorders must be further evaluated.

## AUTHOR CONTRIBUTIONS

M.K.S. and M.A.T. conceived and supervised the study. O.P., L.S.E. and L.M.B. contributed to the study protocol. O.P. and L.S.E. wrote the ethics and Swissmedic applications. L.S.E. and S.M. conducted the study and data collection. S.F. performed image processing and segmentation. L.S.E. analysed the results and conducted the statistical analysis. C.A. shared his clinical expertise as a retinal specialist and helped review the manuscript. L.S.E. primarily wrote the manuscript, with additional support regarding information about the segmentation by S.F. and S.S.‐Z. All authors reviewed the manuscript.

## FUNDING INFORMATION

Research was supported with funds from the research funds of the eye clinic of the cantonal hospital of Lucerne, Switzerland.

## CONFLICT OF INTEREST STATEMENT

All authors, with the exception of S.F. and S.S.‐Z., declare no conflicts of interest. During the initial phase of the conception of this manuscript, S.F. and S.S.‐Z. were employees of EarlySight, with competing interests.

## Supporting information


Figure S1.

Figure S3.

Table S1.

## Data Availability

As this is a clinical trial, the data cannot be shared openly. Upon request to the corresponding author L.S.E., the dataset and code for the statistical analysis can be made available for review.
