# Supplementary material for: Characterization of retinal pigment epithelium layer in healthy and diseased retinas with high‐resolution adaptive optics transscleral flood illumination imaging
Source: Acta Ophthalmol. 2025 Oct 7;104(3):e277–91. doi: 10.1111/aos.70016 (PMC13058683; doi:10.1111/aos.70016)
Supplement: Supplementary file 1 — Figure S1. [file AOS-104-e277-s002.pptx]

## Slide 1
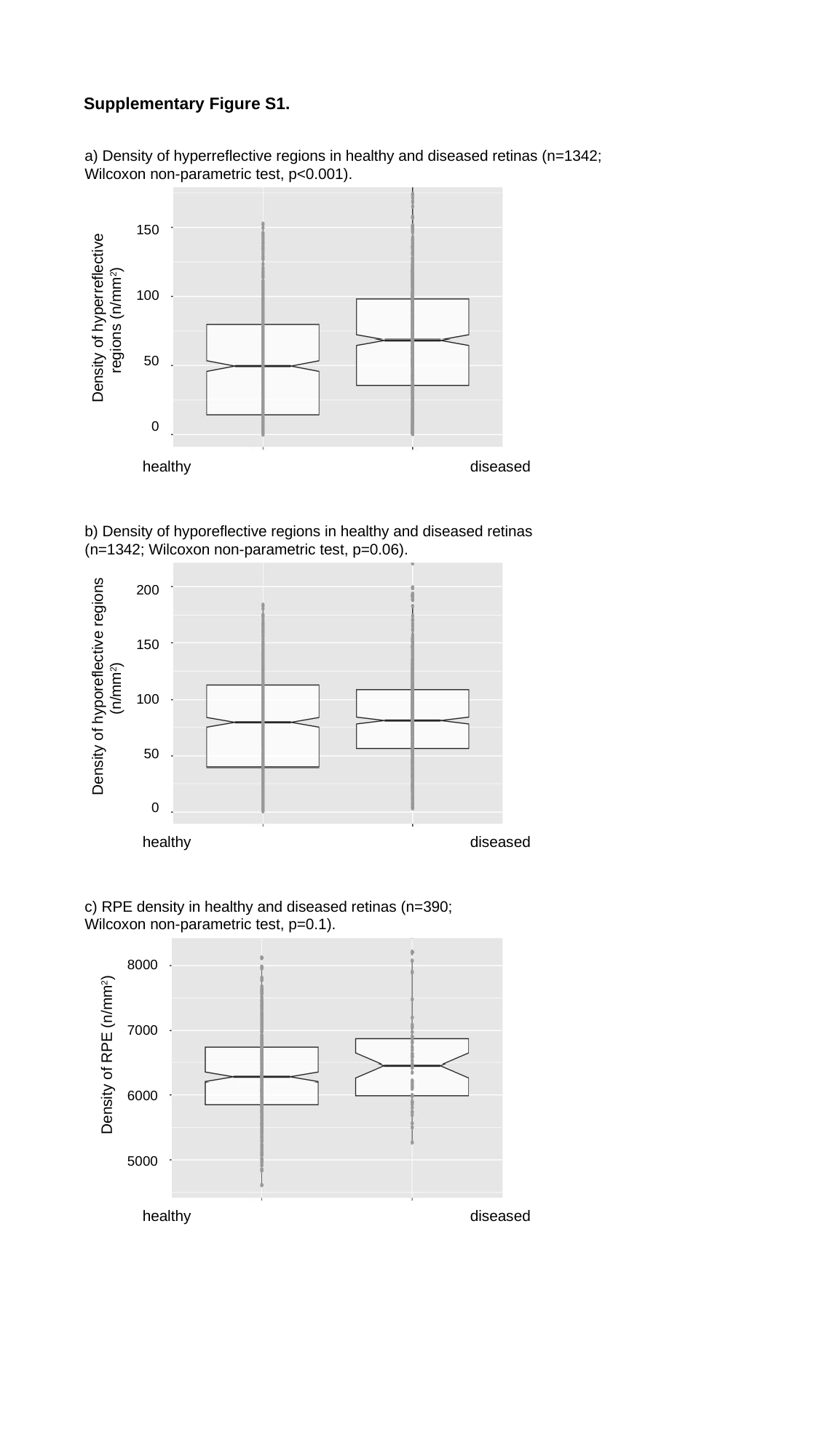

Supplementary Figure S1.
a) Density of hyperreflective regions in healthy and diseased retinas (n=1342; Wilcoxon non-parametric test, p<0.001).
150
100
50
0
Density of hyperreflective regions (n/mm2)
healthy			diseased
b) Density of hyporeflective regions in healthy and diseased retinas (n=1342; Wilcoxon non-parametric test, p=0.06).
200
150
100
50
0
Density of hyporeflective regions (n/mm2)
healthy			diseased
c) RPE density in healthy and diseased retinas (n=390; Wilcoxon non-parametric test, p=0.1).
8000
7000
6000
5000
Density of RPE (n/mm2)
healthy			diseased

## Slide 2
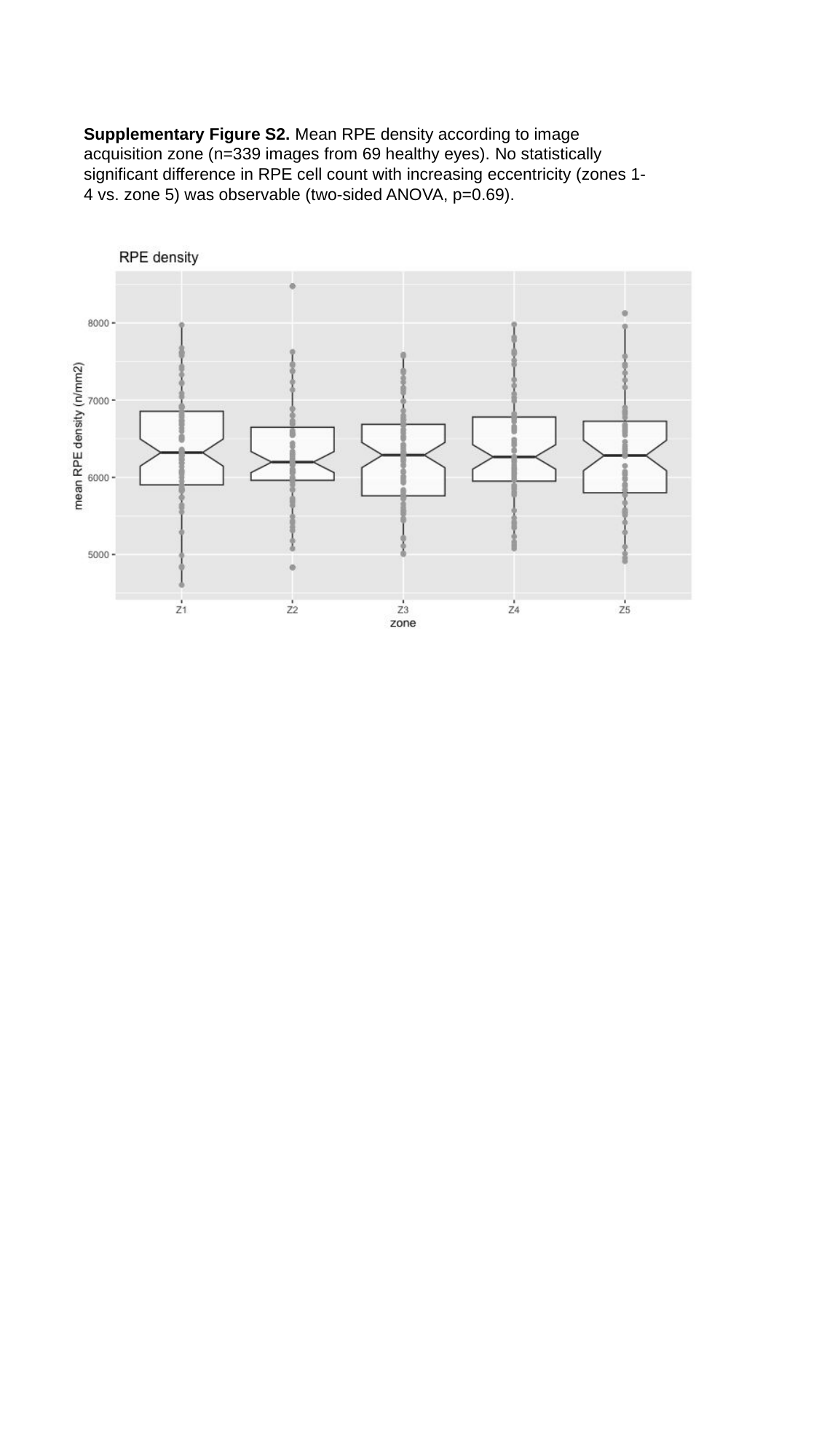

Supplementary Figure S2. Mean RPE density according to image acquisition zone (n=339 images from 69 healthy eyes). No statistically significant difference in RPE cell count with increasing eccentricity (zones 1-4 vs. zone 5) was observable (two-sided ANOVA, p=0.69).
