# Supplementary material for: Characterization of retinal pigment epithelium layer in healthy and diseased retinas with high‐resolution adaptive optics transscleral flood illumination imaging
Source: Acta Ophthalmol. 2025 Oct 7;104(3):e277–91. doi: 10.1111/aos.70016 (PMC13058683; doi:10.1111/aos.70016)
Supplement: Supplementary file 3 — Table S1. [file AOS-104-e277-s003.pptx]

## Slide 1
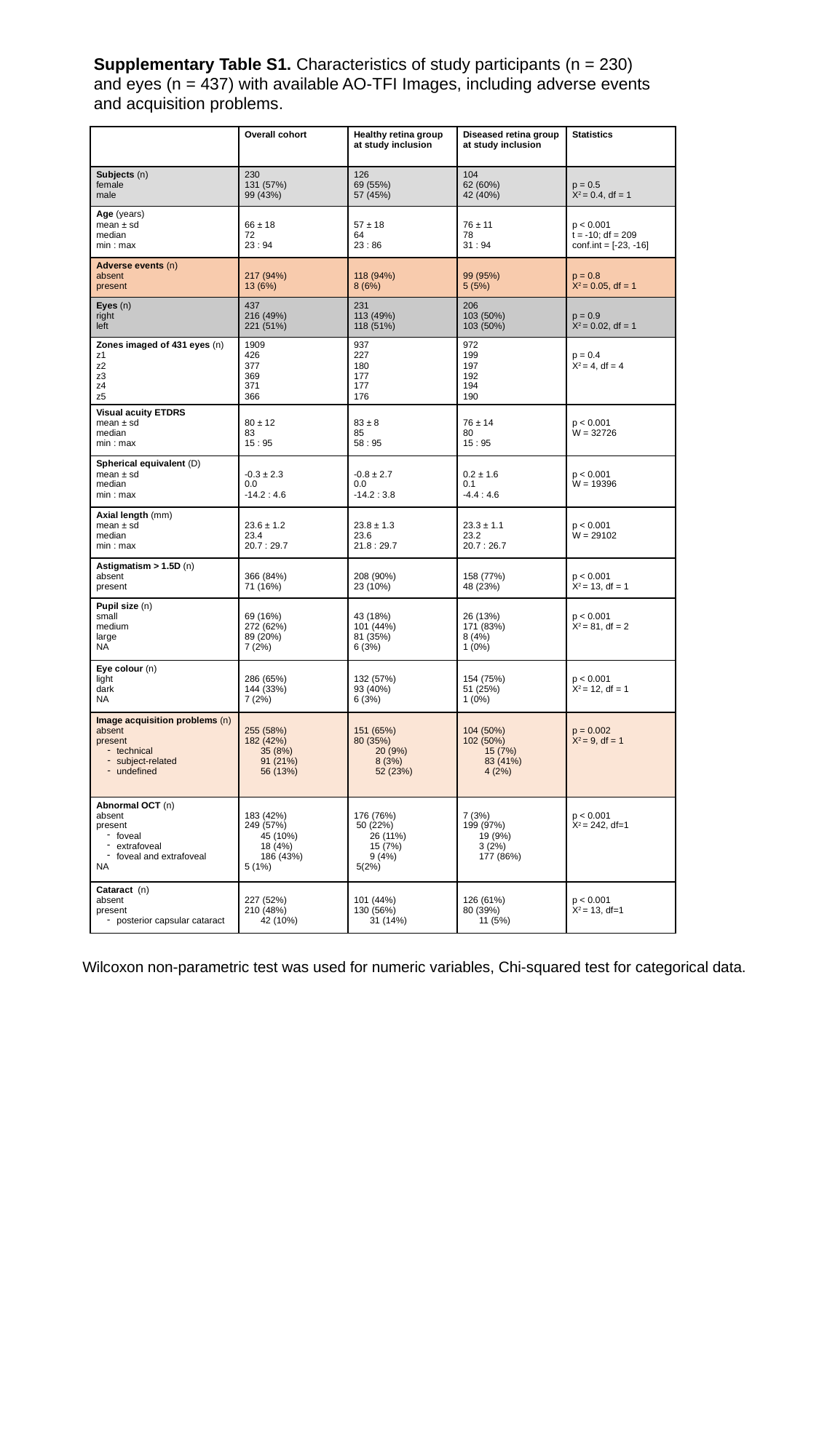

Supplementary Table S1. Characteristics of study participants (n = 230) and eyes (n = 437) with available AO-TFI Images, including adverse events and acquisition problems.
| | Overall cohort | Healthy retina group at study inclusion | Diseased retina group at study inclusion | Statistics |
| --- | --- | --- | --- | --- |
| Subjects (n) female male | 230 131 (57%) 99 (43%) | 126 69 (55%) 57 (45%) | 104 62 (60%) 42 (40%) | p = 0.5 X2 = 0.4, df = 1 |
| Age (years) mean ± sd median min : max | 66 ± 18 72 23 : 94 | 57 ± 18 64 23 : 86 | 76 ± 11 78 31 : 94 | p < 0.001 t = -10; df = 209 conf.int = [-23, -16] |
| Adverse events (n) absent present | 217 (94%) 13 (6%) | 118 (94%) 8 (6%) | 99 (95%) 5 (5%) | p = 0.8 X2 = 0.05, df = 1 |
| Eyes (n) right left | 437 216 (49%) 221 (51%) | 231 113 (49%) 118 (51%) | 206 103 (50%) 103 (50%) | p = 0.9 X2 = 0.02, df = 1 |
| Zones imaged of 431 eyes (n) z1 z2 z3 z4 z5 | 1909 426 377 369 371 366 | 937 227 180 177 177 176 | 972 199 197 192 194 190 | p = 0.4 X2 = 4, df = 4 |
| Visual acuity ETDRS mean ± sd median min : max | 80 ± 12 83 15 : 95 | 83 ± 8 85 58 : 95 | 76 ± 14 80 15 : 95 | p < 0.001 W = 32726 |
| Spherical equivalent (D) mean ± sd median min : max | -0.3 ± 2.3 0.0 -14.2 : 4.6 | -0.8 ± 2.7 0.0 -14.2 : 3.8 | 0.2 ± 1.6 0.1 -4.4 : 4.6 | p < 0.001 W = 19396 |
| Axial length (mm) mean ± sd median min : max | 23.6 ± 1.2 23.4 20.7 : 29.7 | 23.8 ± 1.3 23.6 21.8 : 29.7 | 23.3 ± 1.1 23.2 20.7 : 26.7 | p < 0.001 W = 29102 |
| Astigmatism > 1.5D (n) absent present | 366 (84%) 71 (16%) | 208 (90%) 23 (10%) | 158 (77%) 48 (23%) | p < 0.001 X2 = 13, df = 1 |
| Pupil size (n) small medium large NA | 69 (16%) 272 (62%) 89 (20%) 7 (2%) | 43 (18%) 101 (44%) 81 (35%) 6 (3%) | 26 (13%) 171 (83%) 8 (4%) 1 (0%) | p < 0.001 X2 = 81, df = 2 |
| Eye colour (n) light dark NA | 286 (65%) 144 (33%) 7 (2%) | 132 (57%) 93 (40%) 6 (3%) | 154 (75%) 51 (25%) 1 (0%) | p < 0.001 X2 = 12, df = 1 |
| Image acquisition problems (n) absent present technical subject-related undefined | 255 (58%) 182 (42%) 35 (8%) 91 (21%) 56 (13%) | 151 (65%) 80 (35%) 20 (9%) 8 (3%) 52 (23%) | 104 (50%) 102 (50%) 15 (7%) 83 (41%) 4 (2%) | p = 0.002 X2 = 9, df = 1 |
| Abnormal OCT (n) absent present foveal extrafoveal foveal and extrafoveal NA | 183 (42%) 249 (57%) 45 (10%) 18 (4%) 186 (43%) 5 (1%) | 176 (76%) 50 (22%) 26 (11%) 15 (7%) 9 (4%) 5(2%) | 7 (3%) 199 (97%) 19 (9%) 3 (2%) 177 (86%) | p < 0.001 X2 = 242, df=1 |
| Cataract (n) absent present posterior capsular cataract | 227 (52%) 210 (48%) 42 (10%) | 101 (44%) 130 (56%) 31 (14%) | 126 (61%) 80 (39%) 11 (5%) | p < 0.001 X2 = 13, df=1 |
Wilcoxon non-parametric test was used for numeric variables, Chi-squared test for categorical data.

## Slide 2
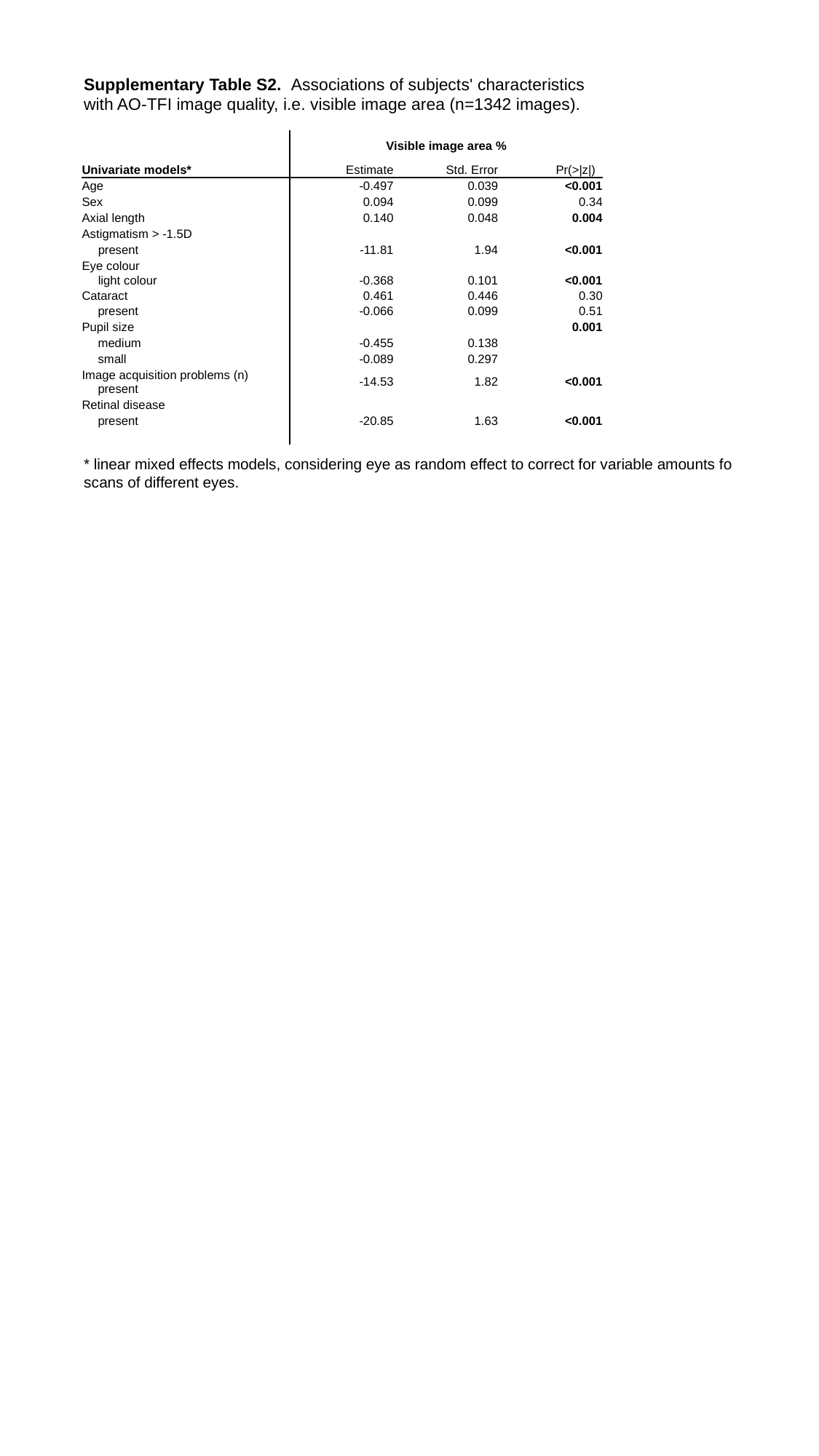

Supplementary Table S2. Associations of subjects' characteristics with AO-TFI image quality, i.e. visible image area (n=1342 images).
| | Visible image area % | | |
| --- | --- | --- | --- |
| Univariate models\* | Estimate | Std. Error | Pr(>|z|) |
| Age | -0.497 | 0.039 | <0.001 |
| Sex | 0.094 | 0.099 | 0.34 |
| Axial length | 0.140 | 0.048 | 0.004 |
| Astigmatism > -1.5D | | | |
| present | -11.81 | 1.94 | <0.001 |
| Eye colour | | | |
| light colour | -0.368 | 0.101 | <0.001 |
| Cataract | 0.461 | 0.446 | 0.30 |
| present | -0.066 | 0.099 | 0.51 |
| Pupil size | | | 0.001 |
| medium | -0.455 | 0.138 | |
| small | -0.089 | 0.297 | |
| Image acquisition problems (n) present | -14.53 | 1.82 | <0.001 |
| Retinal disease | | | |
| present | -20.85 | 1.63 | <0.001 |
| | | | |
* linear mixed effects models, considering eye as random effect to correct for variable amounts fo scans of different eyes.

## Slide 3
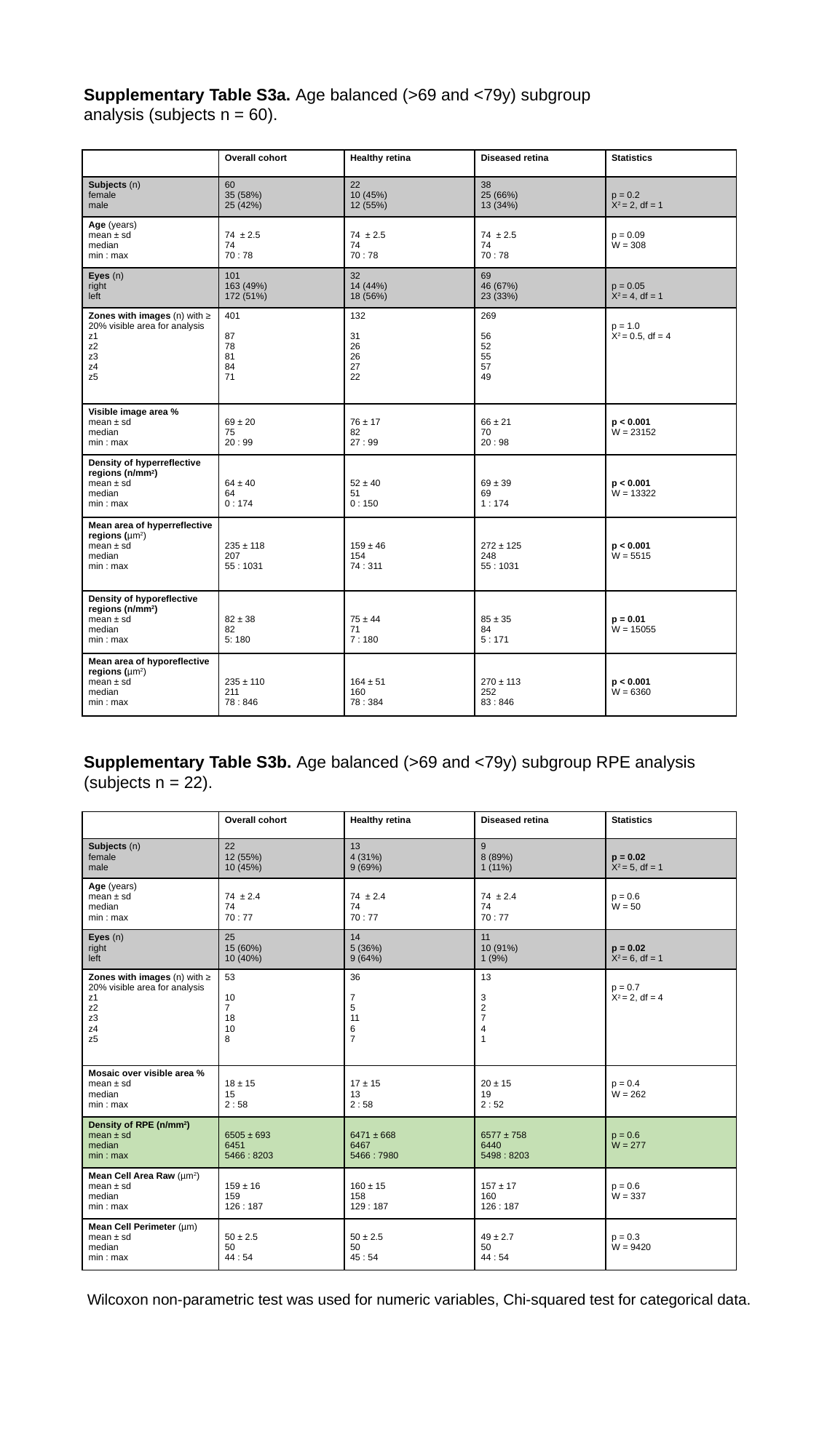

Supplementary Table S3a. Age balanced (>69 and <79y) subgroup analysis (subjects n = 60).
| | Overall cohort | Healthy retina | Diseased retina | Statistics |
| --- | --- | --- | --- | --- |
| Subjects (n) female male | 60 35 (58%) 25 (42%) | 22 10 (45%) 12 (55%) | 38 25 (66%) 13 (34%) | p = 0.2 X2 = 2, df = 1 |
| Age (years) mean ± sd median min : max | 74 ± 2.5 74 70 : 78 | 74 ± 2.5 74 70 : 78 | 74 ± 2.5 74 70 : 78 | p = 0.09 W = 308 |
| Eyes (n) right left | 101 163 (49%) 172 (51%) | 32 14 (44%) 18 (56%) | 69 46 (67%) 23 (33%) | p = 0.05 X2 = 4, df = 1 |
| Zones with images (n) with ≥ 20% visible area for analysis z1 z2 z3 z4 z5 | 401 87 78 81 84 71 | 132 31 26 26 27 22 | 269 56 52 55 57 49 | p = 1.0 X2 = 0.5, df = 4 |
| Visible image area % mean ± sd median min : max | 69 ± 20 75 20 : 99 | 76 ± 17 82 27 : 99 | 66 ± 21 70 20 : 98 | p < 0.001 W = 23152 |
| Density of hyperreflective regions (n/mm2) mean ± sd median min : max | 64 ± 40 64 0 : 174 | 52 ± 40 51 0 : 150 | 69 ± 39 69 1 : 174 | p < 0.001 W = 13322 |
| Mean area of hyperreflective regions (µm2) mean ± sd median min : max | 235 ± 118 207 55 : 1031 | 159 ± 46 154 74 : 311 | 272 ± 125 248 55 : 1031 | p < 0.001 W = 5515 |
| Density of hyporeflective regions (n/mm2) mean ± sd median min : max | 82 ± 38 82 5: 180 | 75 ± 44 71 7 : 180 | 85 ± 35 84 5 : 171 | p = 0.01 W = 15055 |
| Mean area of hyporeflective regions (µm2) mean ± sd median min : max | 235 ± 110 211 78 : 846 | 164 ± 51 160 78 : 384 | 270 ± 113 252 83 : 846 | p < 0.001 W = 6360 |
Supplementary Table S3b. Age balanced (>69 and <79y) subgroup RPE analysis (subjects n = 22).
| | Overall cohort | Healthy retina | Diseased retina | Statistics |
| --- | --- | --- | --- | --- |
| Subjects (n) female male | 22 12 (55%) 10 (45%) | 13 4 (31%) 9 (69%) | 9 8 (89%) 1 (11%) | p = 0.02 X2 = 5, df = 1 |
| Age (years) mean ± sd median min : max | 74 ± 2.4 74 70 : 77 | 74 ± 2.4 74 70 : 77 | 74 ± 2.4 74 70 : 77 | p = 0.6 W = 50 |
| Eyes (n) right left | 25 15 (60%) 10 (40%) | 14 5 (36%) 9 (64%) | 11 10 (91%) 1 (9%) | p = 0.02 X2 = 6, df = 1 |
| Zones with images (n) with ≥ 20% visible area for analysis z1 z2 z3 z4 z5 | 53 10 7 18 10 8 | 36 7 5 11 6 7 | 13 3 2 7 4 1 | p = 0.7 X2 = 2, df = 4 |
| Mosaic over visible area % mean ± sd median min : max | 18 ± 15 15 2 : 58 | 17 ± 15 13 2 : 58 | 20 ± 15 19 2 : 52 | p = 0.4 W = 262 |
| Density of RPE (n/mm2) mean ± sd median min : max | 6505 ± 693 6451 5466 : 8203 | 6471 ± 668 6467 5466 : 7980 | 6577 ± 758 6440 5498 : 8203 | p = 0.6 W = 277 |
| Mean Cell Area Raw (µm2) mean ± sd median min : max | 159 ± 16 159 126 : 187 | 160 ± 15 158 129 : 187 | 157 ± 17 160 126 : 187 | p = 0.6 W = 337 |
| Mean Cell Perimeter (µm) mean ± sd median min : max | 50 ± 2.5 50 44 : 54 | 50 ± 2.5 50 45 : 54 | 49 ± 2.7 50 44 : 54 | p = 0.3 W = 9420 |
Wilcoxon non-parametric test was used for numeric variables, Chi-squared test for categorical data.
